# Supplementary material for: Liquid–Liquid Phase Separation Enhances TDP-43 LCD Aggregation but Delays Seeded Aggregation
Source: Biomolecules. 2021 Apr 8;11(4):548. doi: 10.3390/biom11040548 (PMC8068378; doi:10.3390/biom11040548)
Supplement: Supplementary file 1 [file biomolecules-11-00548-s001.pdf]

Supplementary material

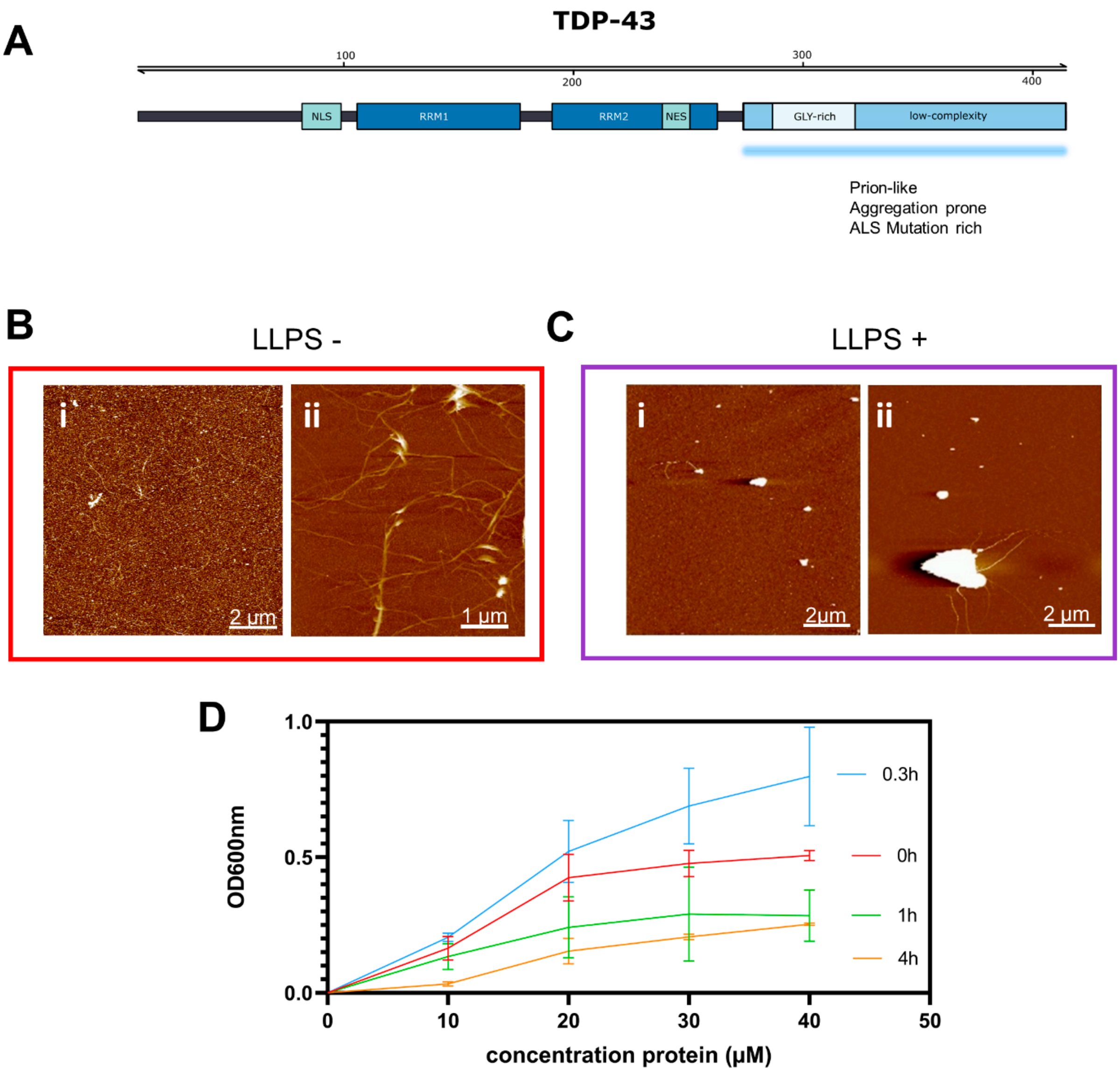

Figure S1. Characteristics of TDP-43 LCD ***(A)*** *Structure of TDP-43 LCD domain, including known domains, characteristics and literature information on the aggregation regions in TDP-43 [1-10]* ***(B,C)*** *AFM images emphasizing difference in morphology of aggregates* ***(B)*** *TDP-43 LCD in LLPS– (i) early time point (0 h) (ii) later time point (0 h)* ***(C)*** *TDP-43 LCD in LLPS+ buffer (i) early time point (0 h) (ii) later time point (4 h) showing two different morphologies.* ***(D)*** *TDP-43 LCD OD600 measurements at varying concentrations at four different time*

points. Based on the graph above, concentrations were selected to study LLPS behavior in this paper. OD is highest at 18 min (0.3 h) after initiation of LLPS. At all time points, the higher the concentration the higher the OD. Mean ± SD shown.

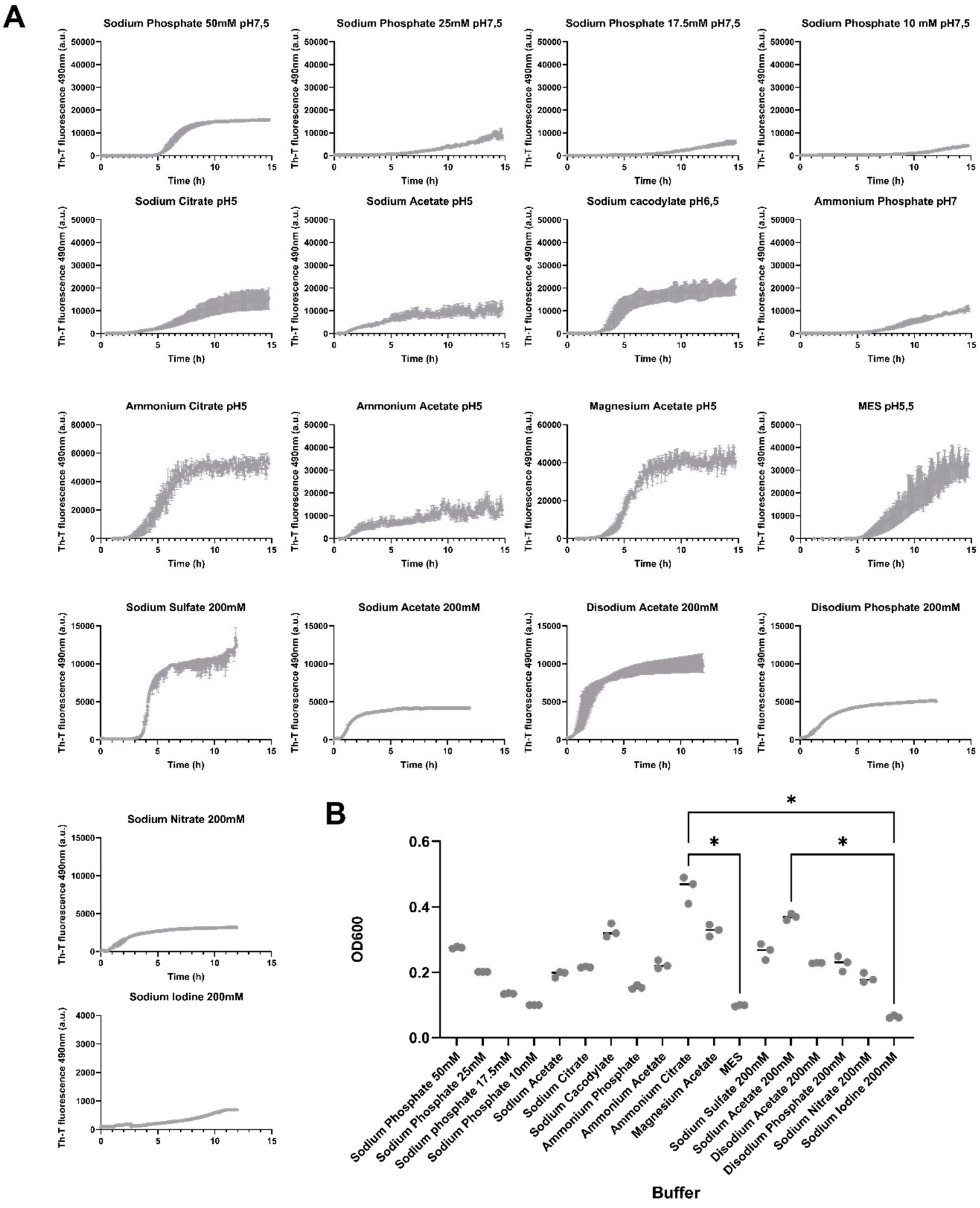

Figure S2 Representation of aggregation and OD600 scores for the buffer screen

(A)*Time course graphs for Th-T aggregation of 18 buffers used in Figure 2, n=3, Mean ± SD is shown.* ***(B)*** *OD600 (at time 0) values determined for all 18 buffers which were used in Table S1 and Figure 2. One-Way ANOVA conducted with Kruskal-Wallis non parametric test for multiple comparisons, n=3, Mean ± SD shown.*

Table S1

Buffer details used in the buffer screen

	Buffer	pH	Conc	K/Ch	*	Mean OD 600 (a.u)	Mean end point aggregation (a.u)	Half time (h)
**1**	Sodium Phosphate	7.5	50mM	K	S	0.276	15747.67	6.2
**2**	Sodium Phosphate	7.5	25mM	K	W	0.203	7931.67	9.7
**3**	Sodium Phosphate	7.5	17.5mM	K	W	0.138	5733.25	12.95
**4**	Sodium Phosphate	7.5	10mM	K	W	0.100	4342.25	13.95
**5**	Sodium Acetate	5	25mM	Ch	W	0.218	15475.75	6.25
**6**	Sodium Citrate	5	25mM	K	S	0.197	20427.5	4.02
**7**	Sodium Cacodylate	6.5	25mM	Ch	W	0.321	10686	4.56
**8**	Ammonium Phosphate	7	25mM	K	W	0.150	10807.33	10.55
**9**	Ammonium Acetate	5	25mM	Ch	W	0.470	12648.33	8.7
**10**	Ammonium Citrate	5	25mM	K	S	0.237	52667.33	4.9
**11**	Magnesium Acetate	5	25mM	K	W	0.342	40529	4.9
**12**	MES ((2-(N-morpholino)ethanesulfonic acid))	6.5	20mM	Ch	W	0.100	32550	9.3
**13**	Sodium Sulfate	7.5	200mM	K	S	0.255	12521.5	4.33
**13**	Sodium Acetate	7.5	200mM	Ch	S	0.370	4191	1.4
**15**	Disodium Acetate	7.5	200mM	K	S	0.225	9722	1.8
**16**	Disodium Phosphate	7.5	200mM	K	S	0.230	5017.93	2.1
**17**	Sodium Nitrate	7.5	200mM	Ch	S	0.190	3142.49	1.8
**18**	Sodium Iodine	7.5	200mM	Ch	S	0.062	691.5	10.4

*** = (W) weak, (S) strong annotation for relative kosmotrope or chaotrope buffer strength**

A

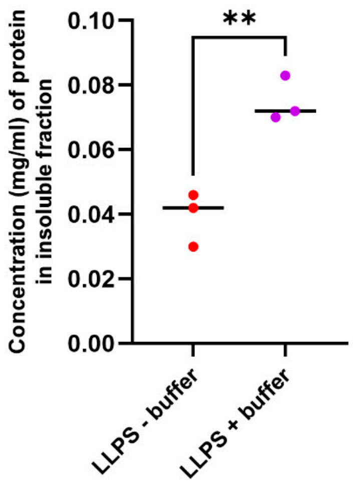

	Percentage (%) of 20μM protein in the insoluble fragment
TDP-43 LCD seed + protein in LLPS - buffer	13%
TDP-43 LCD seed + protein in LLPS + buffer	25%

B

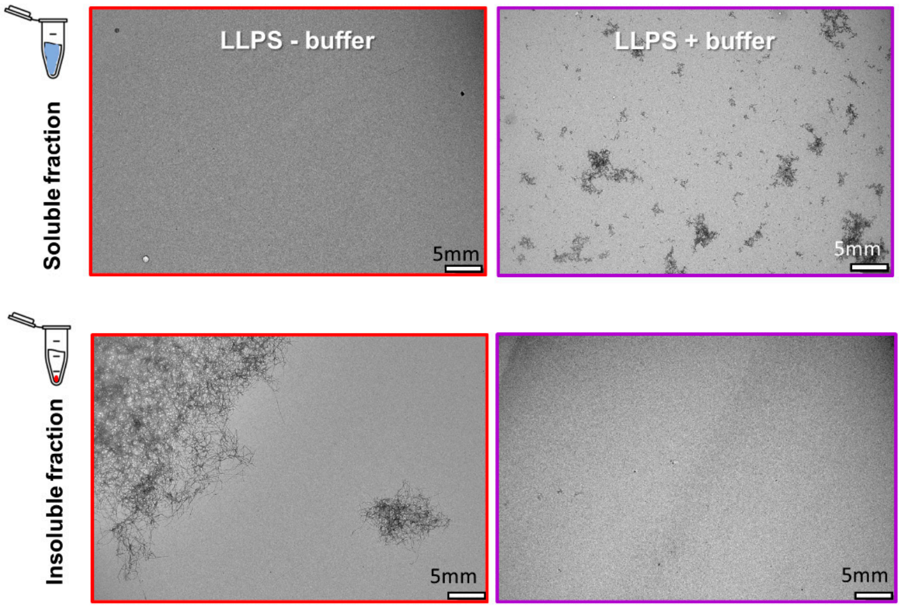

Figure S3 Sedimentation assay related to Figure 4

(A) *Graph showing nanodrop protein concentrations in both LLPS+ and LLPS- buffer after 15 hours with the addition of TDP-43 LCD seeds (1:20 seed to protein molar ration) . Using sedimentation assay, table showing 12% more protein found in solution in LLPS+ buffer than in the LLPS- buffer. This indicates that LLPS+ buffer keeps protein more soluble in a 15 h time frame.* ***(B)*** *EM visualisation of (A) showing insoluble and soluble state of seeding in both buffers. Using centrifugation, more elongated fibrillar protein can be observed in the LLPS– condition in the insoluble state whereas less protein found in LLPS+ buffer, as demonstrated by protein clusters found in the soluble state. Once again, irrespective of seeds, presence of droplets in this system lead to difference in protein morphology*

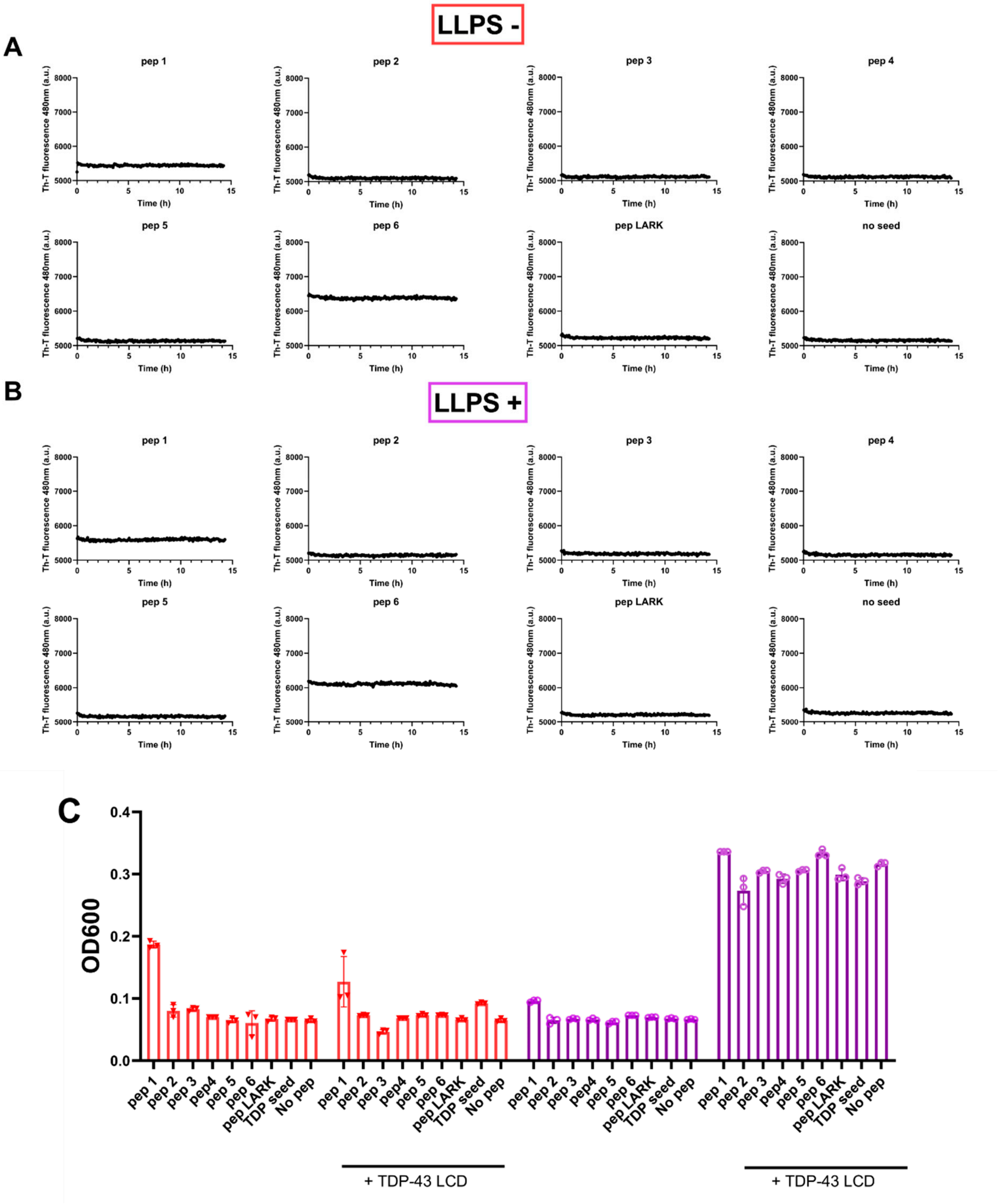

Figure S4 OD600 and ThT graphs for TDP-43 LCD peptides alone in LLPS+ and LLPS- buffers

Corresponding peptide-only (no TDP-43 LCD protein) aggregation curves related to Figure 5B. The peptides alone were not ThT reactive in ***(A)*** *LLPS- conditions or in* ***(B)*** *LLPS+ conditions. Therefore, all effects seen are a result of protein and peptide.* ***(C)*** *OD600*

measurements at time 0, showing that the peptides do not affect TDP-43 LCD turbidity at 1:20 ratio of peptide:protein n=3 ± SD.

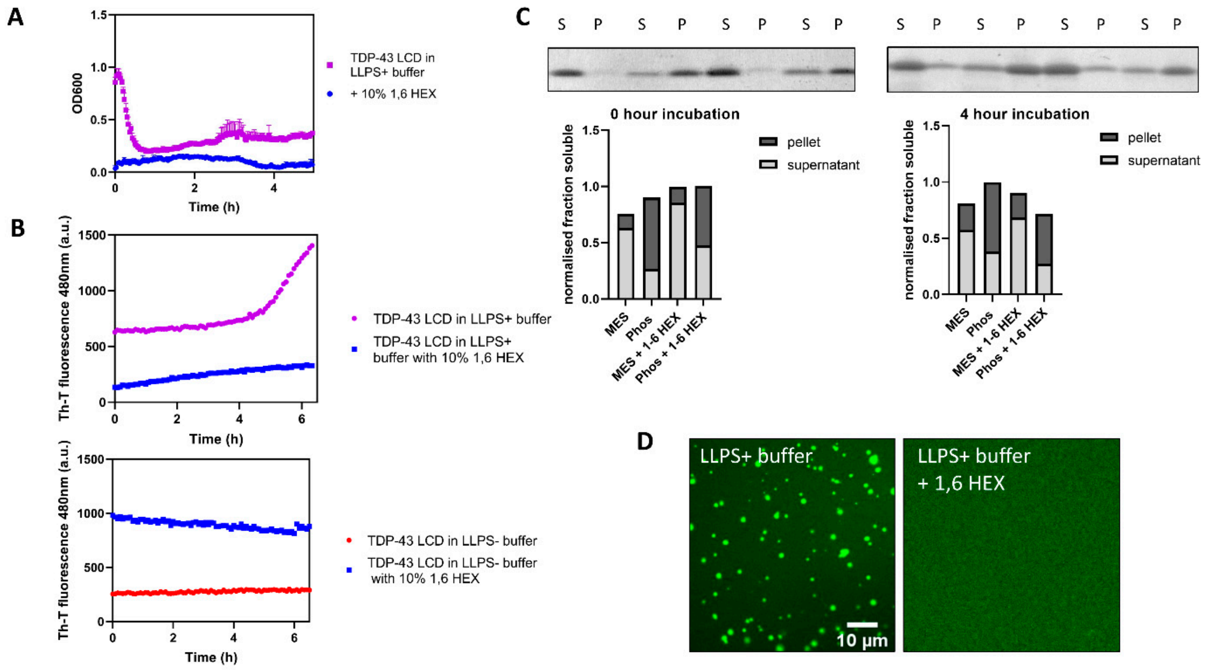

Figure S5 Effect of 1,6-hexanediol on phase separation and solubility of TDP-43 LCD

(A) *Graph showing how 1,6 hexanediol (1,6 HEX) reduces the LLPS behaviour of TDP-43 LCD 20 µM.* ***(B)*** *1,6 HEX reduces the LLPS behaviour of TDP-43 LCD in LLPS buffer, and consequently reduces its ThT value. In MES buffer, 1,6 HEX makes no difference to the kinetics of the aggregation curve.* ***(C)*** *1,6 HEX reduces aggregation of TDP-43 LCD as shown by a reduction of pellet in the LLPS+ and LLPS– condition. A complete removal of pellet was not seen at 4 h indicating that some precipitation still occurs after 1,6 HEX treatment. Analysis based on Coomassie staining of protein.* ***(D)*** *Visualisation using fluorescence microscopy after a 30-min incubation with spiked in labelled TDP-43-LCD protein with and without 1,6 HEX.*

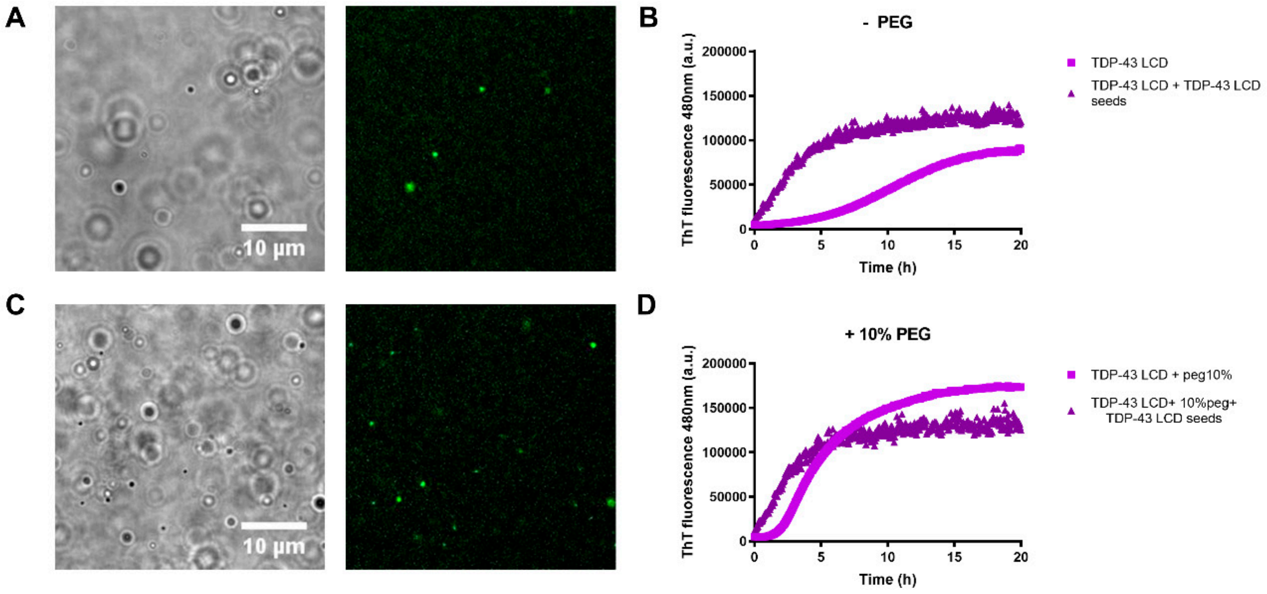

Figure S6 Determination of seeding in the absence and presence of a molecular crowder. *Determination of the ThT fluorescence in the absence or presence of* polyethylene-glycol 4000 (*10% PEG) in phosphate buffer (LLPS+). Visualisation using DIC and fluorescence microscopy after a 5-minute incubation with spiked in labelled TDP-43-LCD protein without (A) and with 10%PEG (C). (B) Seeding can be distinguished between the negative control (no TDP-43 LCD seed) and the condition with TDP-43 LCD seed. However, in the presence of 10% PEG (D), this seeding is not different. Concentration of TDP-43 LCD is 20 µM.*

1. Alexander; Gül; Mittal, J.; Nicolas. ALS Mutations Disrupt Phase Separation Mediated by α-Helical Structure in the TDP-43 Low-Complexity C-Terminal Domain. *Structure* **2016**, *24*, 1537-1549, doi:10.1016/j.str.2016.07.007.
2. Li, H.-R.; Chiang, W.-C.; Chou, P.-C.; Wang, W.-J.; Huang, J.-R. TAR DNA-binding protein 43 (TDP-43) liquid–liquid phase separation is mediated by just a few aromatic residues. *Journal of Biological Chemistry* **2018**, *293*, 6090-6098, doi:10.1074/jbc.ac117.001037.
3. Shimonaka, S.; Nonaka, T.; Suzuki, G.; Hisanaga, S.-I.; Hasegawa, M. Templated Aggregation of TAR DNA-binding Protein of 43 kDa (TDP-43) by Seeding with TDP-43 Peptide Fibrils. *Journal of Biological Chemistry* **2016**, *291*, 8896-8907, doi:10.1074/jbc.m115.713552.
4. Johnson, B.S.; Snead, D.; Lee, J.J.; McCaffery, J.M.; Shorter, J.; Gitler, A.D. TDP-43 Is Intrinsically Aggregation-prone, and Amyotrophic Lateral Sclerosis-linked Mutations Accelerate Aggregation and Increase Toxicity. *Journal of Biological Chemistry* **2009**, *284*, 20329-20339, doi:10.1074/jbc.m109.010264.
5. Colombrita, C.; Zennaro, E.; Fallini, C.; Weber, M.; Sommacal, A.; Buratti, E.; Silani, V.; Ratti, A. TDP-43 is recruited to stress granules in conditions of oxidative insult. *Journal of Neurochemistry* **2009**, *111*, 1051-1061, doi:10.1111/j.1471-4159.2009.06383.x.
6. Saini, A.; Chauhan, V.S. Delineation of the Core Aggregation Sequences of TDP-43 C-Terminal Fragment. *ChemBioChem* **2011**, *12*, 2495-2501, doi:10.1002/cbic.201100427.
7. Dewey, C.M.; Cenik, B.; Sephton, C.F.; Dries, D.R.; Mayer, P.; Good, S.K.; Johnson, B.A.; Herz, J.; Yu, G. TDP-43 Is Directed to Stress Granules by Sorbitol, a Novel Physiological Osmotic and Oxidative Stressor. *Molecular and Cellular Biology* **2011**, *31*, 1098-1108, doi:10.1128/mcb.01279-10.
8. Chen, A.K.H.; Lin, R.Y.Y.; Hsieh, E.Z.J.; Tu, P.-H.; Chen, R.P.Y.; Liao, T.-Y.; Chen, W.; Wang, C.-H.; Huang, J.J.T. Induction of Amyloid Fibrils by the C-Terminal Fragments of TDP-43 in Amyotrophic Lateral Sclerosis. *Journal of the American Chemical Society* **2010**, *132*, 1186-1187, doi:10.1021/ja9066207.
9. Guo, W.; Chen, Y.; Zhou, X.; Kar, A.; Ray, P.; Chen, X.; Rao, E.J.; Yang, M.; Ye, H.; Zhu, L.; et al. An ALS-associated mutation affecting TDP-43 enhances protein aggregation, fibril formation and neurotoxicity. *Nature Structural & Molecular Biology* **2011**, *18*, 822-830, doi:10.1038/nsmb.2053.
10. Cushman, M.; Johnson, B.S.; King, O.D.; Gitler, A.D.; Shorter, J. Prion-like disorders: blurring the divide between transmissibility and infectivity. *Journal of Cell Science* **2010**, *123*, 1191-1201, doi:10.1242/jcs.051672.
